# The Prevalence and Characteristics of Body Dysmorphic Disorder Among Adults in Makkah City, Saudi Arabia. A Cross-Sectional Study

**DOI:** 10.7759/cureus.35316

**Published:** 2023-02-22

**Authors:** Samaa A Sindi, Mohammed k Alghamdi, Eyad E Sindi, Mohammed F Bondagji, Doaa S Baashar, Jihad A Malibary, Mohammad M Alkot

**Affiliations:** 1 Medicine, Umm Al-Qura University, Makkah, SAU; 2 Family Medicine, Umm Al-Qura University, Makkah, SAU

**Keywords:** body dissatisfaction, saudi arabia, makkah, prevalence, body image, body dysmorphic disorder

## Abstract

Background: Body dysmorphic disorder (BDD) is a mental health condition where a person spends much time worrying about flaws in their appearance. The international prevalence of BDD had been reported, and it was about 1.9-2.2%.

Objectives: The current study aims to explore the prevalence of BDD among the general population in Makkah, Saudi Arabia.

Methods: This is a descriptive cross-sectional study that used an electronic questionnaire. It was distributed to the general population using the convenience sample technique between September 2021 to November 2021. BDD was assessed among the participants using an Arabic-validated tool. The sample size was calculated to be 385 participants.

Results: The study included a total of 392 participants. Most of them were female (59.7%), 18-27 years old (62.8%), and had bachelor or post-graduate degrees (67.6%). Among all the included participants, only 28 met the criteria of BDD (7.1%). The BDD population had an equal gender ratio, and most included respondents between 18 and 27 years old (78.6%), college students (60.7%), those with the lowest income level (< 5,000 SR) (78.6%), and who had a normal body mass index (46.4%).

Conclusion: The prevalence of BDD in Makkah, Saudi Arabia, was 7.1%. No significant differences were noticed between BDD and non-BDD groups in age, obesity, and gender.

## Introduction

Body dysmorphic disorder (BDD) is a psychological condition in which the patients have an excessive pathological concern about their appearance in addition to excessive fear of ugliness regarding certain aspects that are considered "not right" or even "not noticeable" by others; it is important to know that in the presence of observable defects on appearance, BDD should not be diagnosed [1]. BDD patients have constant worries, anxiety, and sadness about their looks in a way that causes a significant impairment of function and interferes with their lives [1]. They strongly believe they have a defect in their appearance, which makes them ugly or deformed. As a result, they become stressed [2-4]. Reviewing the literature, the risk factors associated with BDD are currently unclear. Nevertheless, multiple risk factors are hypothesized for the development of BDD, including genetic predisposition and childhood adversity, such as bullying or teasing [2,4]. Moreover, lack of family support or sexual abuse may be non-specific factors, in addition to being more aesthetically sensitive than average [2].

A systematic review conducted in 2016 showed that the estimated prevalence of BDD in the general population is around 1.9-2.2% [5]. Locally in Saudi Arabia, the reported prevalence of BDD among the general population is around 4.2-8.8%, and among female students is 4.4-12.3% [6-9]. Since there is no available up-to-date data concerning the BDD prevalence in Makkah City, Saudi Arabia, to address this, the current study explores the prevalence of body dysmorphic disorder among the general population in Makkah City, Saudi Arabia.

## Materials and methods

Study design, population, and sampling

This descriptive cross-sectional study was conducted in public places (malls, cafes, and gardens) in Makkah City, Saudi Arabia, between September 2021 and November 2021. A convenience sample technique was used to select the participants. We included in our study all adult individuals above 18 years old. The exclusion criteria included non-Arabic speakers and participants with congenital anomalies or dermatological diseases. The minimum sample size required for this study was calculated by OpenEpi version 3.0 [10] in consideration of the following: the population size is about 8,325,304 individuals (according to the General Authority for Statistics), keeping the confidence interval (CI) level at 95%, and considering 50% anticipated frequency. The sample size was calculated to be 385 participants. In case of possible data loss, the total sample size required is 400 participants. However, the final collected data was 392 participants.

Data collection and instrument survey

We used a valid, reliable, simple, understandable, Arabic-based self-report questionnaire that reserves participants' privacy. The questionnaire was distributed to all participants throughout the study using a standard way. Data was collected by giving the participants an electronic iPad containing the questionnaire, designed using Google forms. Informed consent was obtained before filling out the study questionnaire. In addition, the respondents received an iPad device and a request to participate in the study. We grouped the questionnaire's items into four main sections: Section 1 contains the consent form. Section 2 contains questions of the exclusion criteria, such as (do you have a congenital anomaly or dermatological disease); the participants who answered yes to these questions were referred to a submit page and excluded from the study. Section 3 addressed socio-demographic characteristics (e.g., age, gender, and level of education). Section 4 assessed BDD using the Arabic version of the Body Dysmorphic Disorder Questionnaire (BDDQ). Permission to use the BDDQ was with the corresponding author [6], which was a brief, self-reported measure derived from the Diagnostic and Statistical Manual of Mental Disorders, 4th Edition (DSM-IV) diagnostic criteria for BDD [11]. The BDDQ comprises five closed-ended questions asking respondents if their appearance concerns are a source of worry. If yes, how much anguish are they in, or how much this interferes with their social or vocational functioning? The diagnosis is established when a person answers yes to the first two questions, any of the four items of the third question, b or c items of the fourth question, and no to the last question). Section 5 collects information about the participants' concerns, harassment, and psychiatric problems.

Statical analysis

Data was transferred into the Statistical Package for the Social Sciences (SPSS) software, version 26, for statistical analyses. Frequency, percentage, and mean ± SD were used to describe the participants' demographic data, the prevalence of BDD, and participants' concerns, harassment, and psychiatric problems. Crosstabulation was performed between the socio-demographic data among BDD and non-BDD participants based on Pearson's chi-square test. All statistical analyses were performed using two-tailed tests with an alpha error of 0.05.

Ethical considerations

Ethical approval was obtained from the Biomedical Ethics Committee of the Faculty of Medicine at Umm Al-Qura University (UQU), Makkah Al-Mukarramah, Saudi Arabia (approval number AHQP070921).

## Results

Among 392 participants included in the study, only 28 met the criteria of BDD (7.1%). Most BDD participants were between 18 and 27 years old (78.6%), and the gender was distributed equally between males and females. Most participants were Saudis (96.4%), and the most common educational level was a Bachelor's or Post-graduate degree, followed by a high school certificate or below (50% and 46.4%, respectively). Regarding occupation, 60.7% were college students. Most participants earn less than 5000 Saudi Riyals monthly (78.6%). Nearly half of the BDD participants had a normal range of Body Mass Index (BMI) (46.4%). Most BDD individuals spend an average of more than 4 h on their mobile phones daily (75%). There is no significant association between the BDD group or the non-BDD group with socio-demographic variables, as demonstrated in (Table 1).

**Table 1:** Socio-demographic characteristics by body dysmorphic disorder (BDD) status among the included participants (n = 392). * Including: Self-employment, government, or private employee ** Pearson chi-square test or Fisher's exact test as appropriate. The difference is significant at a P value ≤ 0.05. *** BMI: Body Mass Index.

	BDD, N=28 (7.1%)	Non-BDD, N=364 (92.9%)	Total, N= 392 (100%)	P value**
Age in years				0.19
18-27	22 (78.6%)	224 (61.5%)	246 (62.8%)	
28-37	4 (14.3%)	102 (28.0%)	106 (27.0%)	
≥ 38	2 (7.1%)	38 (10.4%)	40 (10.2%)	
Gender				0.28
Male	14 (50.0%)	144 (39.6%)	158 (40.3%)	
Female	14 (50.0%)	220 (60.4%)	234 (59.7%)	
Nationality				0.38
Saudi	27 (96.4%)	334 (91.8%)	361 (92.1%)	
Non-Saudi	1 (3.6%)	30 (8.2%)	31 (7.9%)	
Educational level				0.08
High school certificate or below	13 (46.4%)	97 (26.6%)	110 (28.1%)	
Diploma degree	1 (3.6%)	16 (4.4%)	17 (4.3%)	
Bachelor's or Post-graduate degree	14 (50%)	251 (69%)	265 (67.6%)	
Occupation				0.14
Employee*	9 (32.1%)	163 (44.8%)	172 (43.9%)	
Unemployed	2 (7.1%)	49 (13.5%)	51 (13%)	
College student	17 (60.7%)	152 (41.8%)	169 (43.1%)	
Income				0.13
< 5,000 SR	22 (78.6%)	235 (64.6%)	257 (65.6%)	
5,000 – 9,999 SR	5 (17.9%)	62 (17.0%)	67 (17.1%)	
≥ 10,000	1 (3.6%)	67 (18.4%)	68 (17.4%)	
BMI***				0.07
Underweight (<18.5)	7 (25%)	38 (10.4%)	45 (11.5%)	
Normal range (18.5-24.9)	13 (46.4%)	152 (41.8%)	165 (42.1%)	
Overweight (25.0-29.9)	6 (21.4%)	124 (34.1%)	130 (33.2%)	
Obese (≥ 30)	2 (7.1%)	50 (13.7%)	52 (13.3%)	
Average hours of phone use in one week				0.63
2 hours or less	1 (3.6%)	5 (1.4%)	6 (1.5%)	
3 - 4 hours	6 (21.4%)	72 (19.8%)	78 (19.9%)	
More than 4 hours	21 (75%)	287 (78.8%)	308 (78.6%)	

The detailed criteria of BDD according to DSM-IV among the included participants (n= 392) (Table 2). Exactly 246 of 392 individuals (62.76%) were preoccupied and concerned with their appearance, and the most frequent body part concern was skin (27.1%), and the minor concern was weight (0.2%) (Table 3). Of those preoccupied, 139 wish to think less about their concerns. Out of those who answered yes to the previous two questions, 42 individuals answered (no) to the third question (the question that meant to exclude eating disorders), and of those, a final 28 participants answered (yes) to one of the four questions (Table 2).

**Table 2:** Criteria of Body Dysmorphic Disorder (BDD) among the included participants. * 4a: Has your defect(s) caused you a lot of distress, torment, or pain? 4b: Has your defect(s) significantly interfered with your social life?. 4c: Has your defect(s) significantly interfered with your schoolwork, your job, or your ability to function in your role? 4d: Are there things you avoid because of your defect(s)?

Items	Frequency (%)	Meeting BDD definition
Q1) Are you worried about how you look? (details in Table 3)		246
No	146 (37.24%)	
Yes	246 (62.76%)	
Q2) Do you think about your appearance problems a lot and wish you could think about them less?		139
No	107 (44.50%)	
Yes	139 (56.50%)	
Q3) Is your main concern with how you look that you aren’t thin enough or that you might get too fat?		42
No	42 (30.22%)	
Yes	97 (69.78%)	
Q4) How has this problem with how you look affected your life?		28
No	14 (33.33%)	
Yes (to any of the four questions*)	28 (66.67%)	

**Table 3:** The prevalence of body parts rated as especially unattractive among the general Saudi population included in the study (246).

The body part of concern	Males		Females		Total	
	No.	%	No.	%	No.	%
Overall dissatisfaction	78	49.4%	168	71.8%	246 participants	
Skin	44	27.8%	131	56.0%	175	27.1%
Hair	34	21.5%	90	38.5%	124	19.2%
Nose, mouth, lips, or jaw shape	21	13.3%	50	21.4%	71	11.0%
Billy shape	33	20.9%	76	32.5%	109	16.9%
Buttocks	18	11.4%	42	17.9%	60	9.3%
Breast size or shape	11	7.0%	33	14.1%	44	6.8%
Genitals	8	5.1%	6	2.6%	14	2.2%
Skin color	10	6.3%	21	9.0%	31	4.8%
Hands	10	6.3%	6	2.6%	16	2.5%
Weight	1	0.6%	0	0.0%	1	0.2%

The most reported concern among the respondents was what they think about themself, not how others think about them (46.4%). Additionally, the majority believe that their appearance looks normal to other people (71.4%). When the participants were asked about the standards by which they judged others, most judged others by factors other than their appearance (96.4%). Regarding the psychiatric history of the included participants, only one reported a history of depression, and another reported a history of anxiety disorders after visiting a psychiatrist (Table 4).

**Table 4:** Concerns, harassment, and psychiatric problems among body dysmorphic disorder (BDD) participants (n=28).

Item	Frequency	Percent (%)
To what extent are you primarily concerned about?		
Primary concern is what others think	8	28.6%
Primary concern is what I think	13	46.4%
I do not have a concern	7	25.0%
How much do you believe others think your appearance is abnormal?		
Others think my appearance normal	20	71.4%
Others think my appearance is severely abnormal	8	28.6%
Standards by which you judge others?		
Mainly by appearance	1	3.6%
By other factors (for example: morals, way of thinking, the way by which a person treats)	27	96.4%
Have you ever tried to consult a psychiatrist?		
Yes	2	7.1%
No	26	92.9%
If answered with “yes” to the previous question, what was your diagnosis?		
Depression	1	50.0%
Anxiety	1	50.0%

## Discussion

This is the first study to detect the prevalence of body dysmorphic disorder in the general population of Makkah, Saudi Arabia. The number of participants who met the criteria of BDD was 28 (7.1%). Our prevalence rate is much higher than in Germany, the United States, and Turkey (1.7%, 2.4%, and 6.3%, respectively) [12-14]. The international prevalence of BDD has been reported as 1.9-2.2% [5]. This discrepancy in the BDD prevalence rate could be attributed to the effect of sociocultural differences on body image, for example, between Arab and western populations. Different BDD screening instruments may produce different results [15]. In Saudi Arabia, multiple studies were conducted to assess the prevalence of BDD; the reported prevalence of BDD among the general population is between 4.2% and 8.8% and 4.4-12.3% among female students [6-9]. A study done in the AL-Qassim region of Saudi Arabia on dermatology patients shows that the prevalence of BDD is 18.6% [16]. Most of the participants were young, from 18-27 years old (89.8%), which is consistent with what was published in 2020 by The General Authority of Statistics in Saudi Arabia, in which the majority of the Saudi society is between 15 and 34 years old [17].

The current study revealed an equal distribution between males and females in the BDD population. Compared to previous studies included in a systematic review, there was female predominance with a ratio of 1.27 for the community of adults. In students, there was female predominance, with a ratio of 1.64; however, there was male predominance in cosmetic surgery (with a ratio of 0.71) and rhinoplasty (with a ratio of 0.91) [5].

 Several reports have shown that BDD is associated with obsessive-compulsive disorder (OCD), anorexia nervosa, major depressive disorder (MDD), generalized anxiety disorder (GAD), and social anxiety disorder [18,19]. One study showed a prevalence rate of 12.1% in OCD patients [18]. Our study found one participant with a history of depression and another with a history of anxiety disorders after visiting a psychiatrist.

In comparison, Wei Li et al. found that two BDD patients suffered from comorbid MDD, three suffered from dysthymic disorder, one had panic disorder, one suffered from MDD and GAD, and one suffered from MDD, GAD, and social anxiety disorder [19]. According to the findings, there is no strong association between BDD and other mental disorders.

A strong relationship between body dysmorphic disorder and obesity has been reported in the literature; one exciting finding is that most patients who visited clinics to do abdominoplasty present with mild to moderate BDD, with significant concerns about their weight and shape.

This could be explained by the fact that approximately 70% of the population in the western world is obese [20]. In addition, another study found a significant relationship between obesity/overweight and BDD [9]. However, our study shows an insignificant difference in obesity between BDD and non-BDD groups (p > 0.05). This finding is supported by previous studies, which have suggested that people with no BDD are more concerned about the shape or size of the belly than those with BDD [21]. There are still many unanswered questions about the relationship between obesity and BDD, and further research should be undertaken to investigate the association between BDD and obesity.

This study is the first study conducted among the general population in Makkah. Additionally, BDDQs were collected in an in-person self-reported fashion using an electronic device to minimize the risk of bias.

Limitations

Our study has certain limitations. First, self-report bias is probable since the participants were the only reporters for all study variables, which participants' opinions might influence. Second, the present study used a cross-sectional design which precludes the ability to make causal conclusions. Despite these limitations, our study's findings emphasize the presence of remarkable BDD among the population of Makkah, Saudi Arabia, and clarify the aspects that may need further investigation.

## Conclusions

In this survey, we found the prevalence of BDD patients among the Makkah population to be 7.1%. Most BDD patients were 18-27 years old with no gender predominance. No significant differences were noticed between BDD and non-BDD groups in age, obesity, and gender. Further investigations are needed to demonstrate the characteristics and determinants of BDD.
